# Implementation of an Analytical Model for Leakage Neutron Equivalent Dose in a Proton Radiotherapy Planning System

**DOI:** 10.3390/cancers7010427

**Published:** 2015-03-11

**Authors:** John Eley, Wayne Newhauser, Kenneth Homann, Rebecca Howell, Christopher Schneider, Marco Durante, Christoph Bert

**Affiliations:** 1 Department of Radiation Physics, The University of Texas MD Anderson Cancer Center, 1515 Holcombe Blvd., Houston, TX 77030, USA; E-Mails: jeley@som.umaryland.edu (J.E.); khomann@houstonmethodist.org (K.H.); rhowell@mdanderson.org (R.H.); 2 Graduate School of Biomedical Sciences, The University of Texas, 6767 Bertner Ave., Houston, TX 77030, USA; 3 Department of Physics and Astronomy, Louisiana State University and Agricultural and Mechanical College, 202 Nicholson Hall, Tower Drive, Baton Rouge, LA 70803, USA; E-Mail: cschn19@tigers.lsu.edu; 4 Mary Bird Perkins Cancer Center, 4950 Essen Lane, Baton Rouge, LA 70809, USA; 5 GSI Helmholtzzentrum für Schwerionenforschung, Planckstr. 1, Darmstadt 64291, Germany; E-Mails: m.durante@gsi.de (M.D.); christoph.bert@uk-erlangen.de (C.B.)

**Keywords:** neutron, dose algorithm, equivalent, proton, therapy, stray dose

## Abstract

Equivalent dose from neutrons produced during proton radiotherapy increases the predicted risk of radiogenic late effects. However, out-of-field neutron dose is not taken into account by commercial proton radiotherapy treatment planning systems. The purpose of this study was to demonstrate the feasibility of implementing an analytical model to calculate leakage neutron equivalent dose in a treatment planning system. Passive scattering proton treatment plans were created for a water phantom and for a patient. For both the phantom and patient, the neutron equivalent doses were small but non-negligible and extended far beyond the therapeutic field. The time required for neutron equivalent dose calculation was 1.6 times longer than that required for proton dose calculation, with a total calculation time of less than 1 h on one processor for both treatment plans. Our results demonstrate that it is feasible to predict neutron equivalent dose distributions using an analytical dose algorithm for individual patients with irregular surfaces and internal tissue heterogeneities. Eventually, personalized estimates of neutron equivalent dose to organs far from the treatment field may guide clinicians to create treatment plans that reduce the risk of late effects.

## 1. Introduction

Commercially available proton treatment planning systems (TPSs) typically calculate absorbed dose using pencil beam algorithms that model charged particle radiation transport. Absorbed dose from neutrons is implicitly included in the calculation of dose to tissues inside the therapeutic radiation field; however, neutron equivalent dose outside of the therapy field is usually neglected. This unaccounted for dose has the potential for harm, as neutron exposures are known to contribute to the risk of long-term side effects of proton therapy, including the development of secondary malignant neoplasms (SMNs). Hence, there is a need to develop the capability to predict out-of-field neutron equivalent dose using TPSs.

As cancer screening, diagnosis, and therapy improve, and cancer patients live longer, the potential for late effects also grows. The Surveillance, Epidemiology, and End Results program reports that, as of 2009, over 12 million cancer survivors were living in the United States [1], and approximately one-half of all cancer patients received radiation therapy at some stage of their treatment. The 5-year survival rate of children with all cancers has increased over the past four decades from 60% to nearly 80% [2,3]. For these patients, the cumulative incidence for SMNs 30 years after childhood cancer diagnosis was found to be 7.9% [4]. Given the large and increasing number of cancer survivors who received radiotherapy, there is an urgent need to better understand and mitigate against the risks from stray radiation [5,6].

Measurements indicate that the equivalent dose from neutrons produced from proton treatments is relatively low, typically less than 1% of the prescribed dose, throughout the entire patient [7,8,9,10]. Nevertheless, in organs far from the treatment field, neutrons are the main contributors to equivalent dose, and they, therefore, govern the risk of radiation-related late effects in those organs. To better understand neutron equivalent dose distributions, many groups have conducted Monte Carlo simulation studies [11,12,13,14,15,16,17,18,19,20]. Although Monte Carlo simulations benchmarked against measurements showed good agreement [9,12,14,21,22,23], Monte Carlo simulation of neutron equivalent dose for individual patients is complex and time consuming, requiring hundreds of CPU-hours for a single patient calculation [16,17,24,25]. To allow fast calculation of neutron equivalent dose for individual patients, Zheng *et al.* [26] developed an analytical model by fitting Monte Carlo simulated neutron equivalent dose distributions in air. The model was subsequently extended to account for attenuation of neutrons in water [27] and, later, to explicitly model neutrons characterized as either intranuclear-cascade, evaporation, epithermal, or thermal [28]. Anferov [29] also developed an analytical model to predict equivalent dose from secondary neutrons produced by protons stopping in the treatment nozzle and a phantom. However, these previous studies did not account for individual patient anatomy, *i.e.*, irregular exterior surfaces and internal heterogeneities. Consequently, there is incomplete knowledge of the computational time required to calculate out-of-field secondary neutron dose with an analytical algorithm in clinically realistic, voxelized representations of patient anatomy.

The aims of this study were to demonstrate the feasibility of implementing an analytical model for neutron equivalent dose in a TPS and to estimate the additional computation time required. To accomplish these aims, we implemented an analytical neutron dose model in a research TPS and extended the model to take into account the dosimetric effects of irregular surface contours and heterogeneous patient anatomy. We also compared calculation times using our analytical model with those done using a Monte Carlo simulation model.

## 2. Methods

In this study, we extended the neutron dose equivalent analytical model from Pérez-Andújar *et al.* [28] to take into account individual patient anatomy. We begin our report by briefly reviewing the analytical model (hereafter referred to as the *H/D* model) and then describe our extensions of the *H/D* model to take into account irregular surfaces and internal heterogeneities in a patient. Finally, we describe how we implemented our modified *H/D* model in a TPS and tested its feasibility for calculating neutron equivalent dose from passively scattered proton treatments in a water-box phantom and a voxelized representation of a patient.

### 2.1. Analytical Model for Neutron Equivalent Dose

Neutron equivalent dose, *H*, in water per prescribed proton dose, *D*, (prescribed to a point at isocenter in a water phantom) was modeled following Pérez-Andújar *et al.* [28] as:

(1) $${\left(H/D\right)}_{x,y,z}={\left(H/D\right)}_{\text{iso}}{\left(d/d_{\text{iso}}\right)}^{-p}\sum_{i=1}^{4}C_{i}\;\text{exp}\;[-\alpha_{i}(d^{´}-d_{\text{iso}}^{´})]\;\text{exp}\;[-(x^{2}+y^{2})d_{\text{iso}}^{2}/(2\sigma_{i}^{2}z^{2})]$$

where (*H/D*)_iso_ is the neutron equivalent dose per proton absorbed dose at isocenter, which we determined for modulated proton beams using:

(2) $${\left(H/D\right)}_{\text{iso}}=\frac{1}{n_{\text{max}}}\sum_{j=1}^{N}{\left(H/D\right)}_{\text{iso},j}n_{j}$$

where the proton beam is conceptually divided into *N* finite energy bins, *i.e.*, that cover the range of proton energies incident on the patient; *n*_j_ is the number of protons in the *j*th energy bin, and *n*_max_ is the number of protons in the maximum energy bin. Monoenergetic (*H/D*)_iso,j_ values were taken from Pérez-Andújar *et al.* [28] as well as the additional fitting parameters required (*p*, *C*_i_, α_i_, σ_i_). This model allowed calculation of neutron equivalent dose at a point (*x*, *y*, *z*) that is a distance, *d*, from the virtual neutron source with a path-length of *d*’ in water. For this model, we consider the virtual neutron source to be on central axis on the downstream plane of the patient-specific collimator.

### 2.2. Attenuation of Neutrons Due to Irregular Surfaces and Heterogeneous Tissues

To extend the capability of the *H/D* model to predict neutron dose for voxelized representations of a patient’s anatomy, we used a ray-tracing method [30] to calculate attenuation of the neutron dose distribution in heterogeneous tissues. The voxelized anatomy was derived from computed tomography (CT) images of a patient. The attenuation pathlength term *d*’ in Equation (1) was calculated taking into account irregular patient surfaces and heterogeneous tissue densities. Rays were cast that originated at the virtual neutron source, and they were terminated at the calculation point, e.g., the center of a voxel. The total attenuation pathlength at the calculation point was computed by summing the individual attenuation pathlengths of each traversed voxel.

### 2.3. Implementation of the Analytical H/D Model in a TPS

The extended analytical model for *H/D* was implemented in a research version of the 4D TPS for ion therapy (TRiP) [31,32] using the C programming language. The existing ray-tracing algorithm in TRiP was adapted for the *H/D* model to determine the water-equivalent depth *d*’ at each calculation point as well as the geometric parameters (*x*, *y*, *z*, and *d*) with respect to the central-point at the plane of collimation, where a large majority of neutrons striking the patient originate. These calculations were performed sequentially to find *H/D* for each voxel of the patient CT. The pencil beam dose algorithm in TRiP was used to calculate absorbed dose, *D*, from protons for the passive scattering proton treatment plan. Because TRiP was developed for scanned-beam applications, passive scattering was simulated by creating an artificial planning target volume, PTV_PS_ that mimicked scattered beam delivery. To achieve this, an aperture was defined using the boundary of the original PTV projected to beam’s eye view. Within this aperture, the maximal difference between radiological depths of the proximal and distal edges of the PTV was determined, *i.e.*, the range modulation width of the PTV. To form the PTV_PS_, we included all voxels that were within the aperture and upstream of the distal PTV edge within the range modulation width. Finally, a scanned-beam treatment plan was optimized for the generated PTV_PS_, mimicking a passive scattering treatment plan.

### 2.4. Calculation of Equivalent Dose Distributions from Neutrons in a Water Phantom

A box-shaped water phantom (30 × 15 × 15 cm^3^) was created that contained a spherical target 5 cm in diameter at a depth of 7.5 cm. A single proton treatment field was designed to uniformly irradiate the target. Proton absorbed dose was converted to equivalent dose using a mean radiation weighting factor of 1. Equivalent dose distributions were computed for the water phantom for (1) protons only and (2) the combined contribution from protons and neutrons. Three-dimensional (3D) equivalent dose distributions were visualized using multiple 2D figures of equivalent dose using colorwash and contour plots. Calculation time was recorded for both the proton absorbed dose calculation and the neutron equivalent dose calculation. (*H/D*)_iso_ was 3.68 mSv/Gy for the water phantom plan (*cf.* Equation (2)), using 140 MeV protons, *i.e.*, entering the nozzle, with 5-cm range modulation, and a “medium” scattering foil (specific to the MDACC design).

### 2.5. Calculation of Neutron Equivalent Dose Distribution in a Patient

To test the integration of the *H/D* model, ray tracing algorithm, and TPS, we prepared a treatment plan using voxelized anatomic patient data. For this component of the study, we selected records for a young woman who had received mediastinal irradiation for early stage Hodgkin lymphoma (HL). This patient was selected for two reasons: (1) the treated anatomy provided clinically realistic features that were of relevance to our study, e.g., irregular patient surfaces and internal tissue heterogeneities and (2) expected long survival time and therefore elevated risk of radiogenic late effects. In particular, HL patients undergoing combined radiotherapy and chemotherapy may expect survival rates of approximately 90% at 5 years and higher than 60% at 20 years [33,34]. Importantly, epidemiological studies have revealed that HL patients treated with radiation are at increased risk of SMNs, e.g., cancer in the female breast, lung, and thyroid [35].

The 26-year-old woman in our study had been diagnosed with stage IIB HL and had a solid mediastinal tumor mass. She received passively scattered proton therapy at The University of Texas MD Anderson Cancer Center in Houston, TX (MDACC). Data for this patient were collected under a retrospective research protocol approved by our institutional review board. All protected health information was removed from the electronic medical record using the methods of Newhauser *et al.* [36]. The treatment plan included a single anterior-posterior field to irradiate the tumor using 160 MeV protons entering the nozzle, with 5.5-cm range modulation, 1.5-cm range shifter, and a medium scattering foil. Proton absorbed dose was converted to equivalent dose using a mean radiation weighting factor of 1. Equivalent dose distributions were computed separately for protons and neutrons and summed to find the total equivalent dose distribution. For the HL patient, we applied a field-size correction factor to our model for neutron equivalent dose. Specifically, we multiplied the (*H/D*)_iso_ value of 5.09 mSv/Gy, which was determined for this patient plan using Equation (2), by a factor of 0.623 that represented the ratio of blocked-to-total area of the proton field incident on the patient-specific brass collimator. This accounted for a decrease in neutron production in the treatment head when less collimation is used, needed since the neutron model parameters from Pérez-Andújar *et al.* [28] were determined for protons striking a closed collimator. Equivalent dose distributions were visualized using equivalent dose colorwash planes, contour plots, and organ equivalent-dose volume statistics. To illustrate an out-of-field organ at risk, we calculated the equivalent dose distribution in the thyroid. Calculation time was recorded for both the proton absorbed dose calculation and the neutron equivalent dose calculation.

### 2.6. Comparison of Analytical H/D Model against a Monte Carlo H/D Model

We compared the *H/D* prediction against a Monte Carlo simulation of neutron equivalent dose for the HL patient case described in the previous section. We used MCNPX (version 2.6, Los Alamos National Laboratory, Los Alamos, NM, USA) [37]; and in-house codes [38] to simulate neutron transport and energy deposition for neutrons produced in the MDACC proton treatment nozzle. In our simulation, proton histories were terminated when they crossed a plane separating the treatment nozzle and the patient; thus, we only simulated dose to the patient arising from neutrons produced in the nozzle. The neutron absorbed dose calculated with Monte Carlo was converted to neutron equivalent dose using a mean radiation-weighting factor for neutrons of 8, as determined by Newhauser *et al.* [17] for passively scattered proton treatment of a medulloblastoma patient.

## 3. Results

### 3.1. Equivalent Dose Distributions in a Water-Box Phantom from the Analytical H/D Model

**Figure 1:**
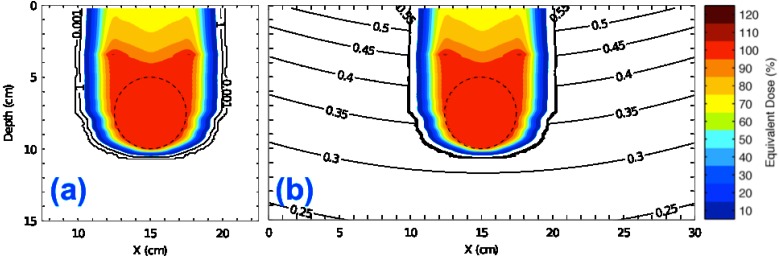
Equivalent dose distributions in planes in the lateral and axial directions for the spherical target in water showing (**a**) proton equivalent dose only and (**b**) combined proton and neutron equivalent dose. Data are shown as percentages of the prescribed target equivalent dose.

The equivalent dose distribution in the water phantom with the spherical target is plotted in Figure 1a. As expected, the proton equivalent dose covered the spherical target uniformly, with a mean equivalent dose for all target voxels of 99.9% of the prescribed dose and a standard deviation of 0.7%. The proton equivalent dose fell off rapidly beyond the lateral target boundary, dropping to almost zero dose (≤0.001%) at 3 cm lateral to the edge of the therapeutic field. Distal to the target, the equivalent dose fell off rapidly to zero within 1 cm when only protons were considered. The sum of the proton and neutron equivalent dose distributions is shown in Figure 1b. The target equivalent dose coverage was nearly identical to that calculated only considering protons. However, the combined equivalent dose distribution extended laterally and distally beyond the target boundary, contributing small but non-negligible equivalent dose (approximately 2–5 mSv/Gy) far (≥10 cm) from the primary field. The proton equivalent dose calculation required 0.20 CPU hours, and the neutron equivalent dose distribution calculation required 0.28 CPU hours.

### 3.2. Equivalent Dose Distributions in a HL Patient from the Analytical H/D Model

**Figure 2:**
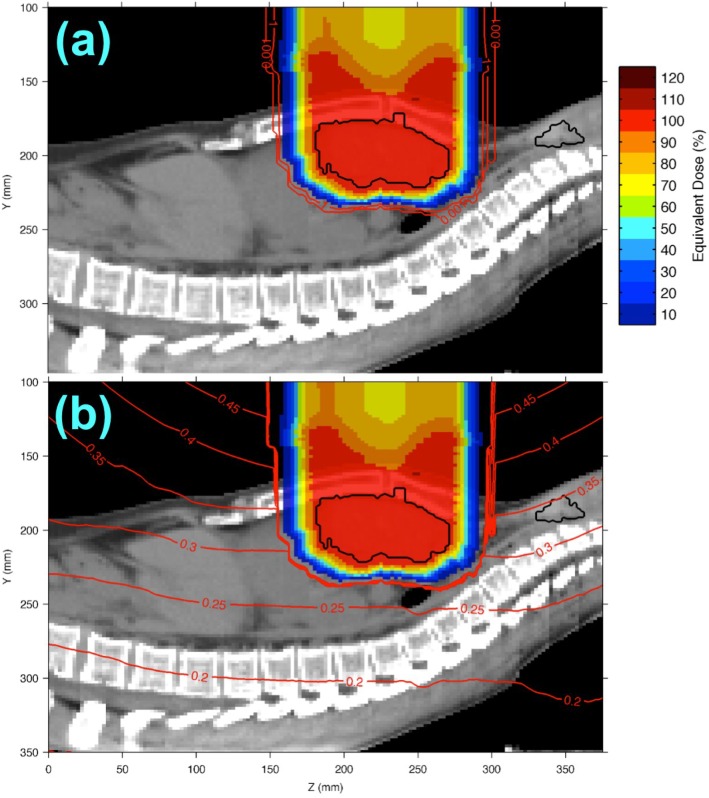
Sagittal equivalent dose planes overlaying a thoracic CT image of the HL patient showing (**a**) proton equivalent dose and (**b**) combined proton and neutron equivalent dose. Equivalent dose values are percentages of the prescribed target equivalent dose, *i.e.*, 36 Sv. The mediastinal tumor and healthy thyroid are contoured in black.

The equivalent dose distribution from a therapeutic proton beam in the HL patient is shown in Figure 2a. A mean equivalent dose of 36 Sv was seen in the target, falling off to zero dose approximately 3.5 cm laterally and 2.5 cm distally. The combined proton and neutron equivalent dose distribution is shown in Figure 2b. Similar to our observations for the water phantom, the target coverage was nearly identical to that of proton dose only. Also consistent with our water phantom observation, the equivalent dose distribution remained between 0.2% and 0.5% of the prescribed dose at large distances (≥15 cm) from the target boundary. For the HL patient, the proton equivalent dose calculation required 0.37 CPU hours, and the neutron equivalent dose calculation required 0.63 CPU hours.

**Figure 3:**
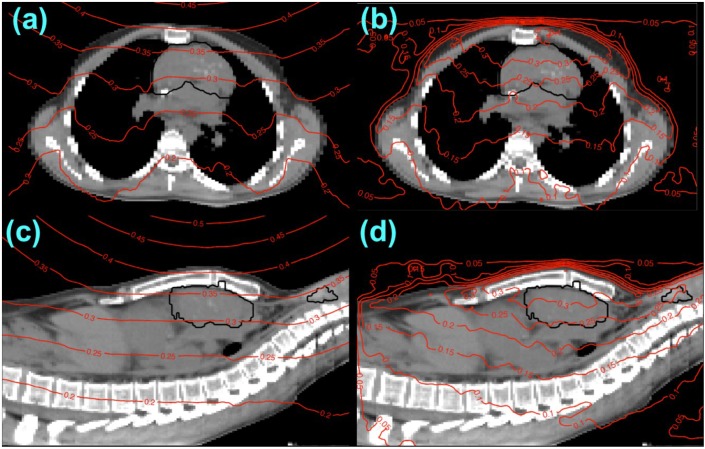
Axial (**a**) and (**b**) and sagittal (**c**) and (**d**) planes of equivalent neutron dose overlaying CT images of the HL patient, with dose calculated using the analytical *H/D* model (**a**) and (**c**) and the Monte Carlo *H/D* model (**b**) and (**d**). Equivalent dose values are percentages of the prescribed target dose. The mediastinal target volume and thyroid are indicated by black contours.

To illustrate the spatial variation in the predicted equivalent dose distribution from neutrons, we plotted the neutron component alone in Figure 3a,c. The isodose contours are seen to diverge from the virtual neutron source, which was located 33 cm upstream of the target. However, variation of the neutron equivalent dose due to irregular patient surfaces and internal density heterogeneities is seen in the overall irregular shape of the neutron isodose contours. In particular, isodose contours extend deeper in lower-density lung regions (Figure 3a), suggesting the importance of using heterogeneity corrections when calculating neutron dose distributions in a clinically realistic setting.

### 3.3. Dose Calculations Using the Analytical Model and Monte Carlo Simulations

Comparing *H/D* values from our analytical model with those from Monte Carlo simulations, we found similar predicted neutron equivalent dose distributions within the HL target of 115 ± 8 mSv and 101 ± 14 mSv for the analytical model (Figure 3a,c) and Monte Carlo simulation (Figure 3b,d), respectively. We observed similar equivalent dose distributions with depth on the central axis inside the patient, but there were more pronounced differences in the overall shape of equivalent dose distributions in the lungs and the air outside the patient, as seen in Figure 3a,b. The analytical *H/D* model predicted a thyroid equivalent dose of 120 ± 3 mSv, *i.e.*, 0.3% of the prescribed dose (Figure 2b). In comparison, the Monte Carlo *H/D* model predicted a thyroid equivalent dose of 86 ± 4 mSv. Our Monte Carlo simulation of neutron equivalent dose required approximately 1600 CPU hours.

## 4. Discussion

We implemented an analytical model to calculate neutron equivalent dose using a proton radiotherapy TPS. We incorporated a ray-tracing algorithm to calculate attenuation of neutron equivalent dose distribution for patients with irregular surface contours and internal heterogeneities. The major finding of this study is that it is feasible to calculate equivalent dose distributions in individual patients as part of the routine treatment planning process.

One of the major clinical implications of this work is that this capability, with further development, might someday enable clinicians to routinely consider quantitative risks of late effects when developing radiotherapy treatment plans. Our approach provides fast neutron calculations, *i.e.*, less than 1 h on a single CPU, a factor of 1.6 times that required for a typical therapeutic proton absorbed dose calculation. It appears straightforward to integrate *H/D* calculations into the current treatment planning workflow. The ability to visualize neutron equivalent dose in commercial TPSs may encourage treatment planning modifications, such as repositioning or shielding of out-of-field organs to reduce exposures without affecting dosimetric coverage of the primary target. Our comparison of the analytical *H/D* model with the more accurate but computationally more expensive Monte Carlo simulations suggests that the two approaches agreed within approximately 15% at the HL tumor isocenter and within 40% at the thyroid. Some of the observed disagreement is likely due to the current approximation that all neutrons diverge from a point in the patient collimator, whereas the more detailed Monte Carlo simulations model the neutron production in detail at multiple places throughout the nozzle, for example in the scattering foils and trimmer-style collimators upstream of the patient collimator.

The current study had several limitations, but none of them prevented us from our primary objective, which was to prove the feasibility of calculating out-of-field neutron dose using an analytical algorithm suitable for inclusion in a current treatment planning process. First, our ray-tracing method, which determined water-equivalent attenuation pathlengths for the neutron distributions, did not consider the elemental composition of the patient but rather used tables designed to convert CT numbers to water-equivalent pathlengths for proton range loss. Thus, the patient was ultimately assumed to consist of voxels having different densities of water. A more physically realistic approach would be to use tables explicitly designed to convert CT numbers to estimate elemental compositions of voxels and ultimately equivalent attenuation pathlengths for neutrons. However, since the *H/D* values from the analytical and Monte Carlo models agreed reasonably well, this approximation appears to have been appropriate for our purposes, though this might also partially explain observed dosimetric discrepancies in air (*cf.* Figure 3) where hydrogen itself is scarce and, thus, recoil-proton dose from neutron collisions is likely lower than predicted when approximating air as very-low-density water. We remark that our Monte Carlo simulations did, in fact, use a CT-to-elemental-composition table as described by Taddei *et al.* [25]. Second, since the vast majority of neutrons are assumed to emanate from plane of final collimation, this method may not be directly applicable to modeling neutrons produced in the patient for a scanned proton therapy system without substantial modification of the model. However, this too is not a serious limitation because the vast majority of proton therapy (>95%) is delivered using passive scattering treatment units. Some of the current limitations of the analytical *H/D* model are being addressed in other studies in our laboratory, including generalization of the model for scanned beam therapy.

## 5. Conclusions

Our results demonstrate that it is feasible to predict neutron equivalent dose distributions using an analytical dose algorithm for individual patients on a timescale comparable to that of routine proton treatment-planning calculations. Eventually, personalized estimates of neutron equivalent dose to organs far from the treatment field may guide clinicians to create treatment plans that reduce the risk of late effects.
